# The effect of physical activity level and exercise training on the association between plasma branched-chain amino acids and intrahepatic lipid content in participants with obesity

**DOI:** 10.1038/s41366-021-00815-4

**Published:** 2021-05-02

**Authors:** Froukje Vanweert, Sebastiaan C. Boone, Bram Brouwers, Dennis O. Mook-Kanamori, Renée de Mutsert, Frits R. Rosendaal, Hildo J. Lamb, Vera B. Schrauwen-Hinderling, Patrick Schrauwen, Matthijs K. C. Hesselink, Esther Phielix

**Affiliations:** 1 Department of Nutrition and Movement Sciences, NUTRIM School of Nutrition and Translational Research in Metabolism, Maastricht University, Maastricht, The Netherlands; 2 Department of Clinical Epidemiology, Leiden University Medical Center, Leiden, The Netherlands; 3 Department of Radiology and Nuclear Medicine, Maastricht University Medical Center, Maastricht, The Netherlands

**Keywords:** Epidemiology, Type 2 diabetes

## Abstract

**Aims:**

To evaluate whether the association between plasma branched-chain amino acids (BCAA) and intrahepatic lipid (IHL) was affected by physical activity level. Furthermore, to investigate if a conventional exercise training program, a subcategory of physical activity, could lower plasma BCAA along with alterations in IHL content in patients with type 2 diabetes (T2DM) and people with nonalcoholic fatty liver (NAFL).

**Methods:**

To investigate the effect of physical activity on the association between plasma BCAA and IHL content, linear regression analyses were performed in 1983 individuals from the Netherlands Epidemiology of Obesity (NEO) stratified by physical activity frequency. Furthermore, the effect of a 12-week supervised combined aerobic resistance-exercise program on plasma BCAA, insulin sensitivity (hyperinsulinemic–euglycemic clamp), and IHL (proton-magnetic resonance spectroscopy (^1^H-MRS)) was investigated in seven patients with T2DM, seven individuals with NAFL and seven BMI-matched control participants (CON).

**Results:**

We observed positive associations between plasma valine, isoleucine and leucine level, and IHL content (1.29 (95% CI: 1.21, 1.38), 1.52 (95% CI: 1.43, 1.61), and 1.54 (95% CI: 1.44, 1.64) times IHL, respectively, per standard deviation of plasma amino acid level). Similar associations were observed in less active versus more active individuals. Exercise training did not change plasma BCAA levels among groups, but reduced IHL content in NAFL (from 11.6 ± 3.0% pre-exercise to 8.1 ± 2.0% post exercise, *p* < 0.05) and CON (from 2.4 ± 0.6% pre-exercise to 1.6 ± 1.4% post exercise, *p* < 0.05), and improved peripheral insulin sensitivity in NAFL as well by ~23% (*p* < 0.05).

**Conclusions:**

The association between plasma BCAA levels and IHL is not affected by physical activity level. Exercise training reduced IHL without affecting plasma BCAA levels in individuals with NAFL and CON. We conclude that exercise training-induced reduction in IHL content is not related to changes in plasma BCAA levels.

**Trial registration:**

Trial registry number: NCT01317576.

## Introduction

Type 2 diabetes patients are characterized by the presence of high levels of branched-chain amino acids (BCAA) in plasma [1–6]. In addition, elevated systemic BCAA levels have been reported prior to the actual onset of type 2 diabetes [5, 7]. Furthermore, the accumulation of plasma BCAA levels is strongly associated with insulin resistance [1–4, 8–11] and mitochondrial dysfunction [12, 13]. It is not known why plasma BCAA are elevated. Some studies relate a higher protein intake that characterize the Western diet, to the increase of BCAA in plasma [2], however, others found no relationship [14, 15]. In addition, it has been suggested that elevated plasma BCAA in individuals with T2DM originates from a blunted inhibitory effect of insulin on proteolysis [16–18] and/or a compromised mitochondrial BCAA metabolism [19–22].

Recently, a cross-sectional study in the Young Finns Study cohort including 338 middle-aged, individuals with overweight/obesity, reported a positive association between plasma BCAA and intrahepatic lipid (IHL) content [23]. Iwasa et al. also described a positive association between plasma BCAA levels and IHL content [24]. In addition, they observed that plasma BCAA levels and fasting glucose levels decreased upon initiation of glucose-lowering therapy [24], but it was not investigated whether the lowering of plasma BCAA levels was associated with a lowering of IHL content. In addition, Kaikkonen et al. showed in a 10-year prospective study that changes in plasma BCAA levels may predict development of nonalcoholic fatty liver (NAFL) in the Young Finns cohort [25]. The mechanism of elevated BCAA levels leading to hepatic fat accumulation is still unknown. However, animal research point toward BCAA-stimulated hepatic lipogenesis via activation of lipogenic genes, such as SREBP-1c, FAS, and ACC [26, 27]. Taken together, we hypothesize that elevated plasma BCAA levels could be a contributing factor to insulin resistance, possibly via modulation of IHL content.

We, as well as others, have previously reported that physical activity and/or exercise training is an effective strategy to improve insulin sensitivity and to reduce IHL content. It is, however, never investigated whether the reduction in IHL relates to a reduction in plasma BCAA levels. Physical activity is defined as movement that require energy expenditure, including walking, cycling sports, and recreation. Exercise training, however, is a subcategory of physical activity that is planned, structured, and repetitive aiming to improve or maintain physical fitness [28]. Whether physical activity and/or exercise affect plasma BCAA levels is still unknown. An observational study showed an association between high physical activity level and low plasma BCAA levels [29]. In contrast, a 12-week endurance and resistance-exercise training study did not result in reduced BCAA levels, while insulin sensitivity did improve [30]. In these studies, IHL content has not been measured.

Based on the strong association between plasma BCAA and IHL content and considering that physical activity level or exercise training may affect both plasma BCAA and IHL, we aimed to investigate whether the association between plasma BCAA and IHL was affected by physical activity level. Furthermore, we investigated if a conventional exercise training program could lower plasma BCAA levels along with alterations in IHL in patients with T2DM and in people with NAFL. We hypothesized that moderate-to-high physical activity and exercise training will result in lower plasma BCAA levels paralleled by a decreased IHL content.

## Materials and methods

### Cross-sectional study

Analyses were performed in 1983 individuals from the Netherlands Epidemiology of Obesity (NEO) study. The NEO study includes middle-aged (45–65 year) individuals, with an oversampling of individuals with overweight and obesity. At baseline, information on demography, lifestyle, and medical history was collected. Physical activity was self-reported using the Short QUestionnaire to ASsess Health enhancing physical activity (SQUASH). In addition, fasting blood samples were collected, and IHL content was quantified by proton-magnetic resonance spectroscopy (^1^H-MRS) on a 1.5-T whole-body scanner (Philips Medical Systems, Best, the Netherlands) as previously described [31]. The data were collected from September 2008 until September 2012 [31]. In 2015, plasma amino acid levels were measured in stored samples (−80 °C) by the Nightingale nuclear magnetic resonance platform and levels were standardized (i.e., mean of zero and standard deviation (SD) of one for each metabolite). The Medical Ethical Committee of the LUMC approved the study, and all participants gave their written informed consent.

### Intervention study

#### Participants

We analyzed BCAA levels in plasma samples derived from the participants previously enrolled in the exercise intervention study as published by Brouwers et al. [32]. In our analysis, we selected seven participants with NAFL, seven patients with T2DM, and seven BMI-matched control participants (CON), all males, based on exercise-induced changes in IHL. Participants had no signs of active cardiac disease, impaired renal or hepatic function [32]. NAFL was defined as having an IHL content of ≥5.0% [33], measured with ^1^H-MRS, and a fasting plasma glucose concentration of <7.0 mmol/l. Patients with T2DM were treated with oral glucose-lowering agents solely and for at least 6 months prior to the onset of the study. Participants were sedentary and maintained their regular dietary behavior throughout the study. Other inclusion criteria were as previously described [32]. All data were collected at the Maastricht University Medical Center, Maastricht, The Netherlands. The study was approved by the local ethics committee and carried out in compliance with the Declaration of Helsinki.

#### Exercise training protocol

The 12-week combined progressive aerobic and resistance-exercise training program, previously performed within our research group [32], included three exercise session per week: two times a week aerobic cycling for 30 min at 70% of maximal work load (*W*_max_) and once a week resistance exercise including three series of ten repetitions at 60% of the maximal voluntary contraction (MVC) with focus at large muscle groups (chest press, leg extension, lat pull down, leg press, triceps curls, biceps curls, abdominal crunched, and horizontal row). MVC was predicted from five multiple repeated maximum testing, as previously described by Reynolds et al. [34]. Total muscle strength was calculated as the sum of the predicted MVC for all eight muscle groups. Warming-up and cooling-down cycling sessions (5 min) were performed at 45% of *W*_max_ before and after every exercise session. The progressive exercise training program was re-assessed for *W*_max_ and MVC after 6 and 4 weeks, respectively, as previously described [35]. Training sessions were performed with three to four individuals at a time.

#### Test day including ^1^H-MRS and two-step clamp

The test days were performed 4 days before the start of the exercise training protocol and between 48 and 72 h after the last exercise bout, as previously reported [32]. In the morning of the test day, all participants underwent ^1^H-MRS on a 3-T whole-body scanner (Achieva 3Tx; Philips Healthcare, Best, The Netherland) to measure IHL content. Then, a primed continuous infusion of [6,6-^2^H_2_]glucose (0.04 mg kg^−2^ min^−1^) was started after which at *t* = 180 min the two-step hyperinsulinemic–euglycemic clamp (10 and 40 mU m^−2^ min^−1^) was started with target glucose value of 5–5.5 mmol/L (Supplementary Fig. 1). The 10 mU m^−2^ min^−1^ insulin infusion step was performed for 4 h to assess hepatic insulin sensitivity and the 40 mU m^−2^ min^−1^ step for 2 h to measure peripheral insulin sensitivity. Steele’s single-pool non-steady state equations were used to calculate glucose rate of appearance (Ra) and glucose rate of disappearance (Rd) [36]. Endogenous glucose production (EGP) was calculated as *R*_*a*_ minus exogenous glucose infusion rate. Hepatic insulin sensitivity was computed as percent insulin-suppressed EGP during the 10 mU m^−2^ min^−1^ low-dose insulin phase. Peripheral insulin sensitivity was measured by insulin-stimulated glucose disposal (*R*_*d*_) under the 40 mU m^−2^ min^−1^ high-dose insulin phase. Due to technical difficulties, ΔRd and insulin-suppressed EGP were determined in six patients with T2DM and six individuals with NAFL. Total plasma aromatic amino acid (AAA) levels were computed as the sum of phenylalanine, tryptophan, and tyrosine level. Total plasma BCAA levels consist of the sum of valine, isoleucine, and leucine level. The insulin-suppressive effect on total plasma BCAA levels (in %) was calculated as the difference in total plasma BCAA levels during the high- and low-dose insulin phase. Further details of the clamp test were previously reported [32].

#### Laboratory analysis

Arterialized blood samples were collected and immediately centrifuged at high speed. Plasma was frozen in liquid nitrogen and stored at −80 °C until assayed. Amino acid levels were determined as previously described [37]. In short, acetonitrile (200 μl) was added to 100-μl plasma for deproteinization, vortex-mixed, and analyzed by liquid chromatography–mass spectrometry for the measurement of concentrated amino acid peaks. This approach, as well as analysis sensitivity, was well validated as previously reported [37]. Calibration curves were constructed to determine the linear ranges of levels.

### Statistics

#### Cross-sectional study

To correct for the oversampling of individuals with a high BMI, all analyses in the NEO study were weighted toward the BMI distribution of the general Dutch population. First, we estimated the difference in amino acid levels between men and women and between a low vs. high physical active group (i.e., less or more than two times per week at least 30 min of moderate intensity) using linear regression analysis. Second, we performed linear regression analyses with fasting plasma amino acid levels as exposure and ln-transformed IHL content as outcome, adjusting for age, sex, total body fat, alcohol intake, energy intake, and leisure time physical activity. In addition, we tested for interaction with sex and physical activity by adding product terms of plasma amino acid levels with sex and physical activity in the regression models. All reported regression coefficients were back-transformed and represent relative changes in IHL content per SD of plasma amino acid levels. Participant characteristics are expressed as mean ± SD for normally distributed data or median with interquartile ranges (IQR) for non-normally distributed data, and differences were assumed to be significant when *p* ≤ 0.05.

#### Exercise intervention study

Baseline comparisons between the three groups were computed with a one-way ANOVA. Linear mixed model analyses were used to analyze differences among groups before and upon exercise. Bonferroni post hoc testing was performed to correct for multiple testing. Pearson correlations were used to evaluate associations between parameters. Sample size calculation was calculated with an *α* of 0.05 and a power of 80% using the formula of correlation sample size (*Z*_0.05_ = 1.9600 and *Z*_0.8_ = 0.8416). Based on the study of Sliz et al. [23], the correlation coefficient is expected to be *r* = 0.57. With the formula, we calculated a sample size of 22 participants. Participant characteristics are presented as mean ± SD. The (insulin-suppressed) plasma amino acids, IHL, Rd, and EGP are presented as mean ± SEM, and differences were assumed to be significant when *p* ≤ 0.05. All analyses were computed with SPSS software for MacOS or Windows.

## Results

### Cross-sectional study

In the cross-sectional analyses, we examined 1983 participants whose characteristics are reported in Supplementary Table 1. The mean plasma levels of isoleucine, leucine, and valine were 50.3 ± 14.7, 66.0 ± 13.7, and 153.9 ± 27.6 μmol/l, respectively, in the 1983 individuals of the NEO study. The correlation coefficients between dietary intake of protein, derived from food frequency questionnaires, and amino acids were close to zero, indicating no relation with plasma levels of isoleucine (*r* = −0.09, *p* = 0.004), leucine (*r* = −0.06, *p* = 0.061), valine (*r* = 0.03, *p* = 0.391), phenylalanine (*r* = 0.03, *p* = 0.414), tyrosine (*r* = −0.05, *p* = 0.120), and histidine (*r* = −0.01, *p* = 0.786). The median IHL content in men was higher than in women (Supplementary Table 1). Median IHL content in individuals who performed 30 min of moderate intensity activity less than two times per week was 3.3% (IQR: 1.6–7.7%) compared with 2.0% (IQR: 1.2–5.6%) in those who performed such physical activity two or more times per week.

On average, individual BCAA concentrations were significantly higher in men than in women (*p* < 0.05 for all three amino acids). Plasma BCAA levels were positively associated with IHL content after adjusting for age, sex, total body fat, alcohol intake, energy intake, and leisure time physical activity. In women, the association between BCAA levels and IHL content was somewhat stronger (*p* value for interaction <0.05 for all three amino acids), than in men (Supplementary Table 2).

Upon stratification by physical activity frequency, levels of isoleucine, leucine, and valine were 51.9 ± 16.2, 67.1 ± 14.8, and 155.0 ± 29.9 μmol/l, respectively, in less active individuals and 48.1 ± 12.3, 64.6 ± 12.1, and 152.3 ± 24.3 μmol/l, respectively, in more active individuals. Levels of isoleucine and leucine were significantly lower (*p* < 0.001 and *p* < 0.005, respectively) in more active individuals compared to less active individuals. The associations between BCAAs and IHL content were similar in individuals with less or more than two times per week at least 30 min of moderate intensity activity (Table 1).

**Table 1:** Relative change in IHL content and 95% confidence intervals per SD of plasma amino acid level in participants of the NEO study (45–65 years), stratified by frequency of physical activity*.

	Physical activity frequency		*p* value for interaction
	<2 times per week (*n* = 1307)	≥2 times per week (*n* = 676)	
Isoleucine	1.52 (1.43, 1.62)	1.49 (1.33, 1.67)	0.698
Leucine	1.54 (1.43, 1.66)	1.53 (1.39, 1.69)	0.945
Valine	1.28 (1.19, 1.37)	1.32 (1.20, 1.46)	0.529
Phenylalanine	1.13 (1.05, 1.22)	1.15 (1.04, 1.27)	0.745
Tyrosine	1.29 (1.21, 1.38)	1.34 (1.21, 1.48)	0.512
Histidine	1.03 (0.97, 1.10)	1.03 (0.93, 1.14)	0.927

Linear regression analysis including standardized fasting plasma BCAA and aromatic amino acids levels as exposure and log-transformed IHL content as outcome, and were weighted toward the BMI distribution of the general population. Model adjusted for age, sex, body fat %, alcohol and energy intake as well as for leisure time physical activity and include an interaction term between the frequency of physical activity and amino acid concentration. The regression coefficients represent relative changes in IHL content per SD of plasma amino acid level. Such ratio, for example 1.2, can be interpreted as 1.2 times IHL content for each extra SD in amino acid concentration, which would reflect an increase in IHL content from, for example, 5–6%.

*A significant interaction with frequency of physical activity (*p* < 0.05).

### Intervention study

#### Baseline characteristics

Baseline characteristics of participants are reported in Supplementary Table 3. The groups did not differ significantly in age, BMI, fasting plasma FFA, aspartate aminotransferase, alanine aminotransferase, glutamyl transferase, high density lipoprotein, C-reactive protein, VO_2max,_ and *W*_max._ Data on compliance and exercise training results were previously reported [32].

**Fig. 1:**
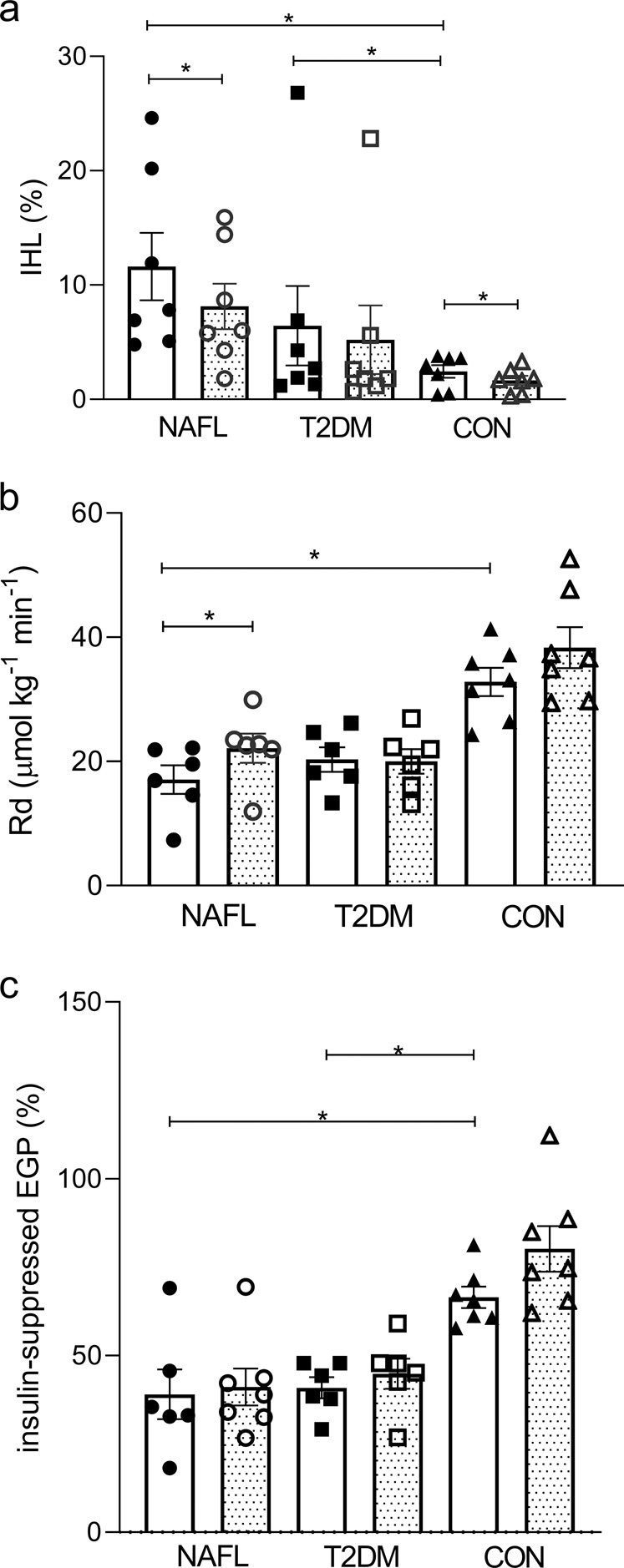
Changes in IHL content, peripheral and hepatic insulin sensitivity after exercise training. IHL content (%) (**a**), peripheral insulin sensitivity expressed as the insulin-stimulated change in glucose uptake (ΔRd μmo kg^−1^ min^−1^) (**b**), and hepatic insulin sensitivity expressed as the insulin-suppressed EGP (%) (**c**) before (open bars) vs. after (dotted bars) exercise training in people with NAFL, T2DM, and CON. IHL was determined in *n* = 7 T2DM, *n* = 7 NAFL, and *n* = 7 CON, however, due to technical difficulties, ΔRd were determined in *n* = 6 T2DM and *n* = 6 NAFL. Data are expressed as mean ± SEM and tested using Anova for repeated measurements; *p < 0.05.

IHL content was significantly different among groups (Anova *p* = 0.012). By design, people with NAFL had high IHL content (≥5%). Patients with T2DM had higher IHL content compared to CON (Fig. 1a, *p* < 0.05). IHL content was not significantly different between NAFL and patients with T2DM (Fig. 1a). Baseline peripheral insulin sensitivity (Fig. 1b) and hepatic insulin sensitivity (Fig. 1c) were also significantly different between groups (Anova *p* < 0.0001 for both). Specifically, they were ~60% and ~38%, respectively, higher in CON compared to NAFL and patients with T2DM (*p* < 0.05 for all). Glucose values throughout the clamp are shown in Supplementary Fig. 1.

**Fig. 2:**
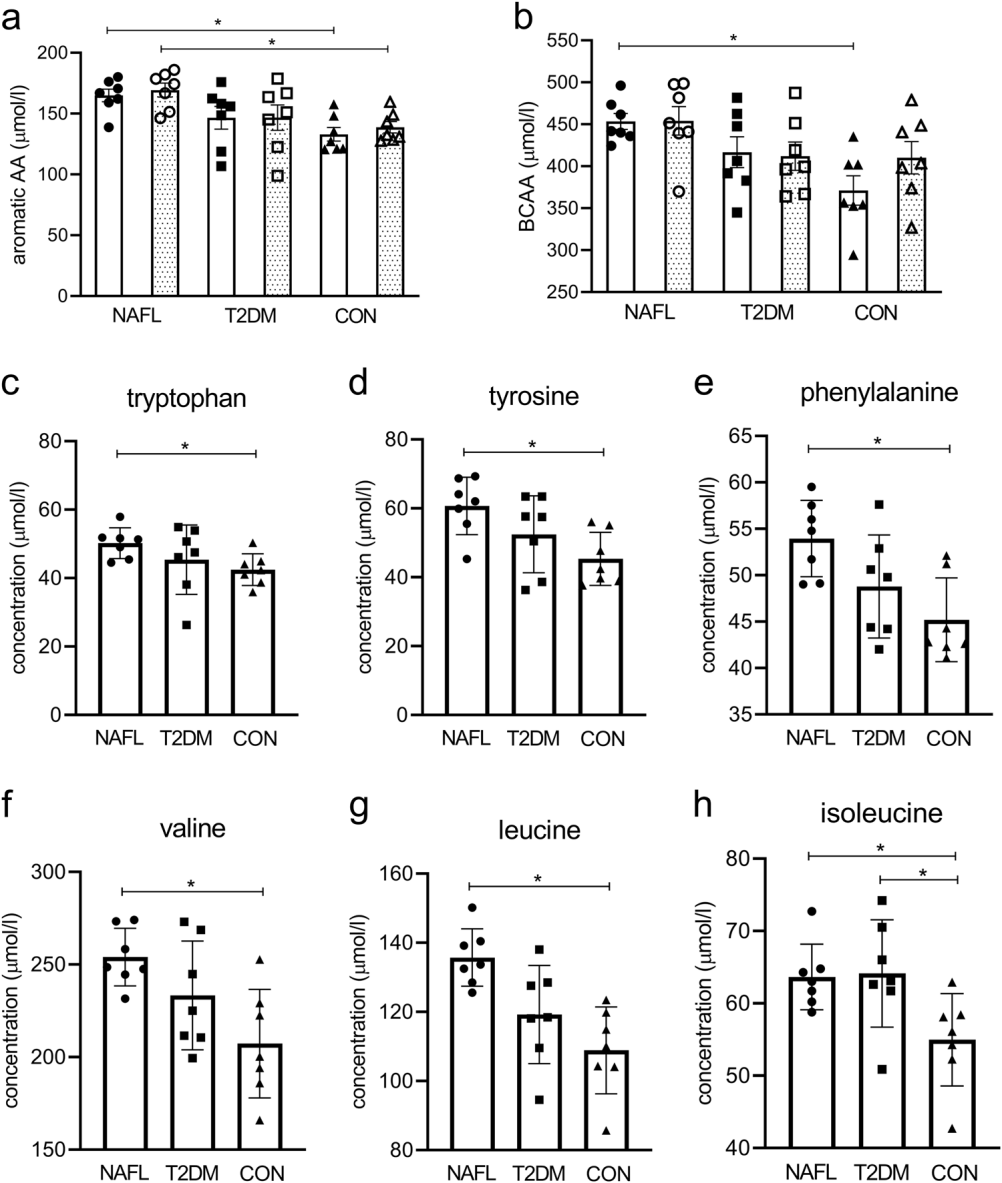
Changes in fasting total AAA and total BCAA levels after exercise training. Fasting total AAA (including phenylalanine, tryptophan, and tyrosine) (**a**) and total BCAA (including leucine, isoleucine, and valine) (**b**) measured in plasma before (open bars) and after (dotted bars) the exercise program. Pre-exercise fasting plasma levels of tryptophan (**c**), tyrosine (**d**), phenylalanine (**e**), valine (**f**), leucine (**g**) and isoleucine (**h**). People with NAFL (dots, *n* = 7), patients with T2DM (squares, *n* = 7), and in CON (triangles, *n* = 7). Data are expressed as mean ± SEM and tested using Anova for repeated measurements; *p < 0.05.

Figure 2 shows fasting plasma levels for total plasma AAA (a) and total BCAA (b) levels in people with NAFL, patients with T2DM and the CON. Data on essential amino acids are shown in Supplementary Fig. 2. Total plasma AAA levels were significantly different among groups (Anova *p* = 0.015), with higher levels in NAFL compared to CON (Fig. 2a, *p* < 0.05). Also, total BCAA levels were significantly different among groups (Anova *p* = 0.006), with significant elevated levels in NAFL compared to CON (Fig. 2b, *p* < 0.05). Of the AAA, plasma tryptophan (Fig. 2c, Anova *p* = 0.05), tyrosine (Fig. 2d, Anova *p* = 0.02), and phenylalanine (Fig. 2e, Anova *p* = 0.01) were all different between groups with higher levels in NAFL compared to CON (*p* < 0.05 for all). Of the BCAA, plasma valine (Fig. 2f, Anova *p* = 0.011), leucine (Fig. 2g, Anova *p* = 0.002), and isoleucine (Fig. 2h, Anova *p* = 0.02) also differed among groups with higher levels in NAFL compared to CON (*p* < 0.05 for all). Plasma isoleucine was also higher in patients with T2DM compared to the CON (Fig. 2h, *p* < 0.05).

#### Associations between fasting plasma BCAA levels, IHL content and insulin sensitivity

**Fig. 3:**
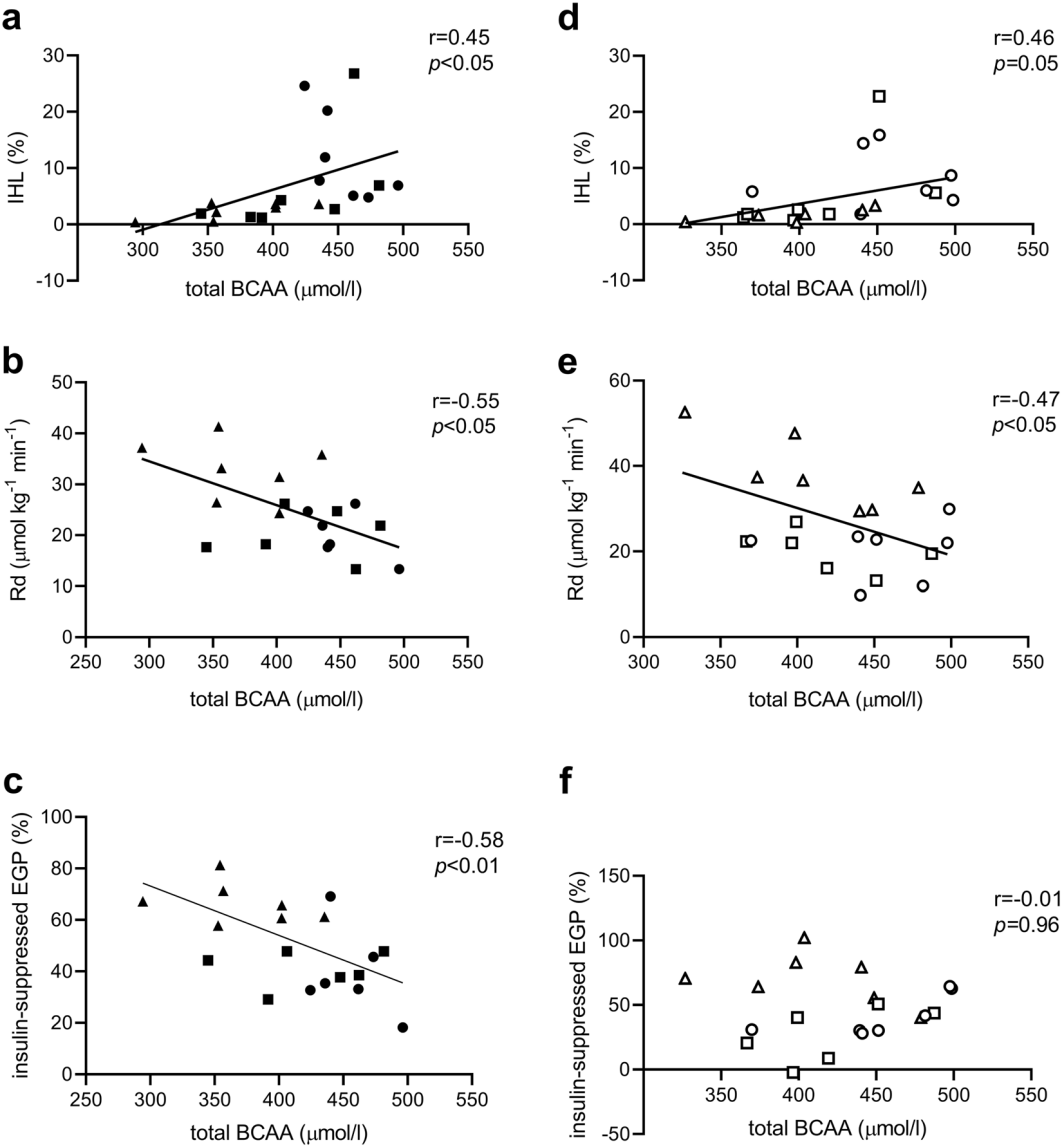
Pearson correlations between fasting total BCAA levels and IHL content, peripheral and hepatic insulin sensitivity. Pre-exercise training (closed symbols): Pearson correlations between plasma BCAA (μmol/l) levels and **a** IHL content (%), **b** peripheral insulin sensitivity expressed as the change in insulin-stimulated glucose disposal (ΔRd μmol kg^−1^ min^−1^), and **c** with the insulin-suppressed EGP (%). Post-exercise (open symbols): correlations between plasma BCAA levels and **d** IHL content, **e** peripheral insulin sensitivity, and **f** with insulin-suppressed EGP (%). Correlations based on the whole group including people with NAFL (dots, *n* = 7), T2DM (squares, *n* = 7), and CON (triangles, *n* = 7), however, due to technical differences only *n* = 6 T2DM and *n* = 6 NAFL were included for the association with ΔRd and insulin-suppressed EGP.

A positive correlation was found between fasting plasma BCAA levels and IHL content (Fig. 3a, *p* < 0.05). Peripheral insulin sensitivity expressed as insulin-stimulated glucose disposal correlated negatively with BCAA levels (Fig. 3b, *p* < 0.05). Furthermore, hepatic insulin sensitivity, expressed as insulin-suppressed EGP, strongly correlated with total BCAA levels (Fig. 3c, *p* < 0.01), as well with all separate BCAA (isoleucine *r* = 0.62, *p* < 0.01, valine *r* = 0.54, *p* < 0.01, and leucine *r* = 0.56, *p* < 0.05). These results indicate that people with high IHL content and those with low peripheral and hepatic insulin sensitivity feature highest plasma BCAA levels.

#### Differences in the insulin-suppressive effect on plasma BCAA levels among groups

**Fig. 4:**
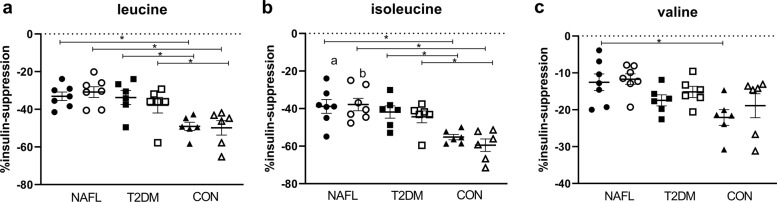
Changes in the insulin-suppressive effect on fasting BCAA levels after exercise training. The percentage of insulin-suppressed leucine (**a**), isoleucine (**b**), and valine (**c**) measured in fasting plasma before (solid symbols) and after exercise training (open symbols) in people with NAFL (NAFL, dots), people with patients with type 2 diabetes (T2DM, squares) and control participants (CON, triangles). Due to technical difficulties, the percentage of insulin-suppressed leucine was determined in *n* = 6 T2DM, *n* = 6 NAFL, and *n* = 7 CON. Data are expressed as mean ± SEM and tested using Anova for repeated measurements; *p < 0.05.

During the clamp, the insulin-suppressive effect on plasma BCAA levels was determined as the change of plasma BCAA levels during the high insulin phase vs. levels measured at basal, expressed as percentage suppression. Insulin suppression for isoleucine (Fig. 4a) and leucine (Fig. 4b) differed between groups (Anova *p* = 0.004 and *p* = 0.001, respectively) and was larger in CON compared to both NAFL and patients with T2DM (*p* < 0.05 for both). Insulin suppression of valine (Fig. 4c) differed as well between groups (Anova *p* = 0.01), with lower suppression values seen in NAFL vs. CON (*p* < 0.05).

#### Exercise training does not affect AAA and BCAA levels

Exercise training had no effect on total plasma AAA (Fig. 2a) or total BCAA (Fig. 2b) levels in NAFL, patients with T2DM, or in CON. Also when groups were pooled, we did not observe exercise-induced changes in total AAA (148 ± 5-μmol/l pre-exercise vs. 152 ± 5-μmol/l post exercise) neither in total BCAA (414 ± 11-μmol/l pre-exercise vs. 425 ± 11-μmol/l post exercise). However, upon exercise, BCAA levels between the groups lost significance (Supplementary Fig. 3a). These absent differences in BCAA concentration between groups upon exercise training were not caused by altered levels in T2DM or NAFL, but due to a slight, nonsignificant increase in the CON (Supplementary Fig. 3b–e).

#### Exercise-mediated effects on IHL content and insulin sensitivity in people with NAFL

Exercise training mediated a reduction in IHL content in NAFL (*p* < 0.05) and in CON (*p* < 0.05), but not in patients with T2DM (Fig. 1a, *p* value Anova repeated measures <0.0001). Exercise training improved peripheral insulin sensitivity in NAFL (*p* < 0.05), but not in people with T2DM nor in CON (Fig. 1b, *p* value Anova repeated measures = 0.019). Hepatic insulin sensitivity did not significantly change with exercise training (Fig. 1c, Anova *p* value for repeated measures 0.086). After exercise training, the associations between plasma BCAA levels with IHL content (Fig. 3d, *p* = 0.05) and peripheral insulin sensitivity (Fig. 3e, *p* < 0.05) persisted; however, no correlation was seen between plasma BCAA levels and peripheral insulin sensitivity (Fig. 3f, *p* = 0.96).

#### Exercise training did not improve the suppressive effect of insulin on plasma BCAA levels

Exercise training had no effect on the insulin-suppressive effect on plasma leucine (Fig. 4a), isoleucine (Fig. 4b), and valine (Fig. 4c) in patients with T2DM, individuals with NAFL, nor in CON. After exercise training, the insulin-suppressive effect on plasma BCAA levels remained highest in CON compared to both NAFL and patients with T2DM (*p* < 0.05 for both).

## Discussion

We here aimed to explore the relationship between plasma BCAA levels and IHL content based on data from a Dutch population-based study and a 12-week exercise intervention program performed in patients with T2DM, NAFL, and CON. We hypothesized that a conventional exercise training program lowers plasma BCAA levels along with alterations in IHL content in patients with T2DM and in people with NAFL. Our results indicate that the association between plasma BCAA levels and IHL content is not affected by physical activity level and/or exercise training. Exercise training reduced IHL content without affecting plasma BCAA levels in individuals with NAFL and CON. We conclude that exercise training does not change plasma BCAA levels, despite reductions in IHL content.

In the present study, we aimed to investigate the relationship between plasma BCAA levels and IHL content and therefore compared amino acid levels between people with NAFL, patients with T2DM and a BMI-matched CON. We found that both AAA and BCAA levels were higher in people with NAFL, who had the highest IHL content, compared to both T2DM and CON. In line with this data, a recent study demonstrated elevated plasma BCAA levels in people with NAFL and concluded that elevated plasma BCAA levels play a role in the progression from NAFL toward the development of type 2 diabetes [38]. Therefore, it seems plausible that both elevated plasma BCAA levels and IHL content may impact insulin sensitivity.

Currently, it is not known why plasma BCAA levels are elevated in insulin-resistant people, in patients with T2DM or in people with NAFL. In theory, a higher protein intake that characterize the Western diet, combined with a mismatch in BCAA oxidation could result in an accumulation of BCAA in plasma. There are reports of a self-administered food frequency questionnaire, protein consumption seemed to be slightly higher in people with obesity when compared to lean participants [2]. Others reported that the overall dietary pattern, rather than the dietary intake of BCAA per se, contributes to high BCAA plasma concentrations thereby modulating the risk for chronic diseases [14, 39]. Importantly, using protein intake derived from food frequency questionnaires in the Dutch NEO cohort showed no relation between dietary intake of protein and BCAA levels. Several reports point however toward diminished or altered function of the key enzymes involved in BCAA catabolism, indicating that disturbances in BCAA metabolism could underlie the elevated plasma levels observed [19–22]. Since insulin inhibits amino acid release by muscle, insulin resistance toward this inhibition may underlie higher plasma BCAA levels in patients with T2DM and humans with NAFL [40]. In line, results from the present study showed a ~15% reduced peripheral suppression of plasma BCAA levels under hyperinsulinemic conditions in both patients with T2DM and in people with NAFL when compared to CON. Whether the resistance toward insulin suppression of plasma BCAA indeed contributes to elevated levels seen upon an overnight fast, cannot be deduced from the current study.

In the present study, we observed a positive association between plasma BCAA levels and IHL content in 1983 participants of a Dutch population-based study. This is in in line with the results of a cross-sectional analysis in the Young Finns Study cohort including 338 middle-aged individuals with overweight and obesity [23]. The association between plasma BCAA levels and IHL content was also supported in a smaller group of people who participated in the exercise intervention study. Whether elevated plasma BCAA contribute to the development of NAFL cannot be concluded from this study. A large prospective study of Kaikkonen et al. [25] points out that higher plasma BCAA levels precede the onset of NAFL in healthy young individuals [25]. Therefore, it is plausible that elevated plasma BCAA levels could contribute to a high IHL content. In line, a recent observational study evaluated relations of plasma BCAA levels and NAFL with incident type 2 diabetes [38]. It was found that elevated BCAA levels in part mediated the associations between NAFL and type 2 diabetes. This suggests that both elevated BCAA levels and IHL content play a role in type 2 diabetes development [38]; however, causality cannot be concluded from the present association analysis and needs to be further investigated.

We found that physically active people (≥2 times per week moderate intensity exercise for at least 30 min) had slightly, but significant lower plasma BCAA levels. The level of physical activity did not alter the positive association between plasma BCAA levels and IHL content. Also, we found that a conventional exercise program including both resistance and endurance training was effective in lowering IHL content in NAFL and CON, but did not coincide with lower plasma BCAA levels. Moreover, the association between IHL content and plasma BCAA levels persisted upon exercise training, albeit less strong. The exercise intervention program did also not induce changes in plasma AAA levels. In the NEO cohort, we investigated the long-term effects of physical activity on plasma BCAA levels, while the 12-week exercise training represents the short-term exercise-induced changes. Based on our results, we can conclude that long-term physical activity leads to a slightly reduction in plasma BCAA levels, but the exercise training showed no short-term changes. In line with our results, an observational study showed an association between high physical activity level and low plasma BCAA levels [29], although an exercise intervention study reported that plasma BCAA levels in insulin-resistant individuals did not reduce after a 12-week endurance resistance training program [30]. These results indicate that exercise training or physical activity differently affect plasma BCAA levels, but not the relation between plasma BCAA and IHL content and suggest that BCAA do not play a role in mediating the beneficial metabolic effects of exercise training on, among others, IHL content. The mechanism of long-term physical activity leading to lower plasma BCAA levels is, however, unknown and needs further research.

A limitation of the present study is that physical activity in the observational study was self-reported with use of the SQUASH. The main limitation of these questionnaire is that individuals may overestimate their true rate of activity, although, SQUASH is a previously validated tool to assess physical activity in the Dutch population [41, 42]. Nevertheless, in our manuscript, we stratified participants into high and low physical activity and therefore we do not expect potential overreporting would have led to misclassification in these groups. In the exercise intervention study, we were not able to investigate gender-specific effects, which, however, is important. In these kinds of invasive intervention studies, unfortunately only a small number of participants can be included; however, this study had enough power to pick up changes in insulin sensitivity and changes in IHL, which were the main objectives of the study.

To summarize, we showed an association between plasma BCAA levels and IHL content in a large Dutch populations-based study, which remained similar in strata of physical activity frequency. Levels of isoleucine and leucine were lower in more active individuals compared to less active individuals. The present study furthermore extends the finding of elevated plasma BCAA levels in people with NAFL as well as the positive association with IHL content, which was not affected after a conventional exercise training program. The exercise training program did not decrease BCAA levels, neither was able to overcome diminished insulin suppression of BCAA levels in people with or without NAFL and/or T2DM. Moreover, our results showed that lower IHL content and improved hepatic insulin sensitivity in people with NAFL upon the exercise training did not coincide with reduced BCAA levels. We conclude that BCAA levels and IHL are associated, but differently affected by physical activity and/or exercise training.

## Supplementary information

Supplementary Information

## Data Availability

All data were analyzed in a blinded fashion. The datasets generated during and/or analyzed during the study are available from the corresponding author on reasonable request.

## References

[1] Huffman KM, Shah SH, Stevens RD, Bain JR, Muehlbauer M, Slentz CA (2009). Relationships between circulating metabolic intermediates and insulin action in overweight to obese, inactive men and women. Diabetes Care.

[2] Newgard CB, An J, Bain JR, Muehlbauer MJ, Stevens RD, Lien LF (2009). A branched-chain amino acid-related metabolic signature that differentiates obese and lean humans and contributes to insulin resistance. Cell Metab.

[3] Palmer ND, Stevens RD, Antinozzi PA, Anderson A, Bergman RN, Wagenknecht LE (2015). Metabolomic profile associated with insulin resistance and conversion to diabetes in the Insulin Resistance Atherosclerosis Study. J Clin Endocrinol Metab.

[4] Tai ES, Tan ML, Stevens RD, Low YL, Muehlbauer MJ, Goh DL (2010). Insulin resistance is associated with a metabolic profile of altered protein metabolism in Chinese and Asian-Indian men. Diabetologia.

[5] Wang TJ, Larson MG, Vasan RS, Cheng S, Rhee EP, McCabe E (2011). Metabolite profiles and the risk of developing diabetes. Nat Med.

[6] Xu F, Tavintharan S, Sum CF, Woon K, Lim SC, Ong CN (2013). Metabolic signature shift in type 2 diabetes mellitus revealed by mass spectrometry-based metabolomics. J Clin Endocrinol Metab.

[7] Floegel A, Stefan N, Yu Z, Muhlenbruch K, Drogan D, Joost HG (2013). Identification of serum metabolites associated with risk of type 2 diabetes using a targeted metabolomic approach. Diabetes.

[8] Perng W, Gillman MW, Fleisch AF, Michalek RD, Watkins SM, Isganaitis E (2014). Metabolomic profiles and childhood obesity. Obesity.

[9] Shah SH, Crosslin DR, Haynes CS, Nelson S, Turer CB, Stevens RD (2012). Branched-chain amino acid levels are associated with improvement in insulin resistance with weight loss. Diabetologia.

[10] Walford GA, Ma Y, Clish C, Florez JC, Wang TJ, Gerszten RE (2016). Metabolite profiles of diabetes incidence and intervention response in the Diabetes Prevention Program. Diabetes.

[11] Wurtz P, Soininen P, Kangas AJ, Ronnemaa T, Lehtimaki T, Kahonen M (2013). Branched-chain and aromatic amino acids are predictors of insulin resistance in young adults. Diabetes Care.

[12] Koves TR, Ussher JR, Noland RC, Slentz D, Mosedale M, Ilkayeva O (2008). Mitochondrial overload and incomplete fatty acid oxidation contribute to skeletal muscle insulin resistance. Cell Metab.

[13] Muoio DM, Newgard CB (2008). Molecular and metabolic mechanisms of insulin resistance and beta-cell failure in type 2 diabetes. Nat Rev Mol Cell Bio.

[14] Merz B, Frommherz L, Rist MJ, Kulling SE, Bub A, Watzl B. Dietary pattern and plasma BCAA-variations in healthy men and women—results from the KarMeN Study. Nutrients. 2018;10:623. 10.3390/nu10050623 PMC5985475 29762522

[15] Lopez AM, Noriega LG, Diaz M, Torres N, Tovar AR (2015). Plasma branched-chain and aromatic amino acid concentration after ingestion of an urban or rural diet in rural Mexican women. BMC Obes.

[16] Mahendran Y, Jonsson A, Have CT, Allin KH, Witte DR, Jorgensen ME (2017). Genetic evidence of a causal effect of insulin resistance on branched-chain amino acid levels. Diabetologia.

[17] Welle S, Nair KS (1990). Failure of glyburide and insulin treatment to decrease leucine flux in obese type II diabetic patients. Int J Obes.

[18] Halvatsiotis P, Short KR, Bigelow M, Nair KS (2002). Synthesis rate of muscle proteins, muscle functions, and amino acid kinetics in type 2 diabetes. Diabetes.

[19] Doisaki M, Katano Y, Nakano I, Hirooka Y, Itoh A, Ishigami M (2010). Regulation of hepatic branched-chain alpha-keto acid dehydrogenase kinase in a rat model for type 2 diabetes mellitus at different stages of the disease. Biochem Biophys Res Commun.

[20] She P, Van Horn C, Reid T, Hutson SM, Cooney RN, Lynch CJ (2007). Obesity-related elevations in plasma leucine are associated with alterations in enzymes involved in branched-chain amino acid metabolism. Am J Physiol Endocrinol Metab.

[21] Adams SH (2011). Emerging perspectives on essential amino acid metabolism in obesity and the insulin-resistant state. Adv Nutr.

[22] Zhou M, Shao J, Wu CY, Shu L, Dong W, Liu Y (2019). Targeting BCAA catabolism to treat obesity-associated insulin resistance. Diabetes.

[23] Sliz E, Sebert S, Wurtz P, Kangas AJ, Soininen P, Lehtimaki T (2018). NAFLD risk alleles in PNPLA3, TM6SF2, GCKR and LYPLAL1 show divergent metabolic effects. Hum Mol Genet.

[24] Iwasa M, Ishihara T, Mifuji-Moroka R, Fujita N, Kobayashi Y, Hasegawa H (2015). Elevation of branched-chain amino acid levels in diabetes and NAFL and changes with antidiabetic drug treatment. Obes Res Clin Pract.

[25] Kaikkonen JE, Wurtz P, Suomela E, Lehtovirta M, Kangas AJ, Jula A (2017). Metabolic profiling of fatty liver in young and middle-aged adults: cross-sectional and prospective analyses of the Young Finns Study. Hepatology.

[26] Du Y, Meng Q, Zhang Q, Guo F (2012). Isoleucine or valine deprivation stimulates fat loss via increasing energy expenditure and regulating lipid metabolism in WAT. Amino Acids.

[27] Cheng Y, Meng Q, Wang C, Li H, Huang Z, Chen S (2010). Leucine deprivation decreases fat mass by stimulation of lipolysis in white adipose tissue and upregulation of uncoupling protein 1 (UCP1) in brown adipose tissue. Diabetes.

[28] Caspersen CJ, Powell KE, Christenson GM (1985). Physical activity, exercise, and physical fitness: definitions and distinctions for health-related research. Public Health Rep.

[29] Kujala UM, Peltonen M, Laine MK, Kaprio J, Heinonen OJ, Sundvall J (2016). Branched-chain amino acid levels are related with surrogates of disturbed lipid metabolism among older men. Front Med.

[30] Glynn EL, Piner LW, Huffman KM, Slentz CA, Elliot-Penry L, AbouAssi H (2015). Impact of combined resistance and aerobic exercise training on branched-chain amino acid turnover, glycine metabolism and insulin sensitivity in overweight humans. Diabetologia.

[31] de Mutsert R, den Heijer M, Rabelink TJ, Smit JW, Romijn JA, Jukema JW (2013). The Netherlands Epidemiology of Obesity (NEO) study: study design and data collection. Eur J Epidemiol.

[32] Brouwers B, Schrauwen-Hinderling VB, Jelenik T, Gemmink A, Sparks LM, Havekes B (2018). Exercise training reduces intrahepatic lipid content in people with and people without nonalcoholic fatty liver. Am J Physiol Endocrinol Metab.

[33] Neuschwander-Tetri BA (2005). Nonalcoholic steatohepatitis and the metabolic syndrome. Am J Med Sci.

[34] Reynolds JM, Gordon TJ, Robergs RA (2006). Prediction of one repetition maximum strength from multiple repetition maximum testing and anthropometry. J Strength Cond Res.

[35] Meex RC, Schrauwen-Hinderling VB, Moonen-Kornips E, Schaart G, Mensink M, Phielix E (2010). Restoration of muscle mitochondrial function and metabolic flexibility in type 2 diabetes by exercise training is paralleled by increased myocellular fat storage and improved insulin sensitivity. Diabetes.

[36] Steele R (1959). Influences of glucose loading and of injected insulin on hepatic glucose output. Ann N Y Acad Sci.

[37] van Eijk HM, Wijnands KA, Bessems BA, Olde Damink SW, Dejong CH, Poeze M (2012). High sensitivity measurement of amino acid isotope enrichment using liquid chromatography-mass spectrometry. J Chromatogr B Analyt Technol Biomed Life Sci.

[38] van den Berg EH, Flores-Guerrero JL, Gruppen EG, de Borst MH, Wolak-Dinsmore J, Connelly MA, et al. Non-alcoholic fatty liver disease and risk of incident type 2 diabetes: role of circulating branched-chain amino acids. Nutrients. 2019;11:705. 10.3390/nu11030705 PMC6471562 30917546

[39] Lynch CJ, Adams SH (2014). Branched-chain amino acids in metabolic signalling and insulin resistance. Nat Rev Endocrinol.

[40] Adeva MM, Calvino J, Souto G, Donapetry C (2012). Insulin resistance and the metabolism of branched-chain amino acids in humans. Amino Acids.

[41] de Hollander EL, Zwart L, de Vries SI, Wendel-Vos W (2012). The SQUASH was a more valid tool than the OBiN for categorizing adults according to the Dutch physical activity and the combined guideline. J Clin Epidemiol.

[42] Wendel-Vos GC, Schuit AJ, Saris WH, Kromhout D (2003). Reproducibility and relative validity of the short questionnaire to assess health-enhancing physical activity. J Clin Epidemiol.

