# Supplementary material for: The effect of physical activity level and exercise training on the association between plasma branched-chain amino acids and intrahepatic lipid content in participants with obesity
Source: Int J Obes (Lond). 2021 May 2;45(7):1510–20. doi: 10.1038/s41366-021-00815-4 (PMC8236400; doi:10.1038/s41366-021-00815-4)
Supplement: Supplementary file 1 — Supplementary Information [file 41366_2021_815_MOESM1_ESM.docx]

**Supplementary Appendix**

Table 1: Characteristics of the NEO study population

|  | **Total** (n=1 983) | **Men** (n=1 042) | **Women** (n=941) |
| --- | --- | --- | --- |
| **Age (years)** | 56 (50-61) | 56 (50-61) | 55 (51-60) |
| **Sex (% men)** | 47.3 | - | - |
| **BMI (kg/m^2^)** | 25.9 ± 3.8 | 26.6 ± 3.4 | 25.3 ± 4.0 |
| **FM (%)** | 30.4 (23.8-37.0) | 23.9 (21.2-27.9) | 36.1 (31.8-40.6) |
| **IHL content (%)** | 2.73 (1.36-6.34) | 3.81 (2.03-8.66) | 1.82 (1.09-4.55) |
| **Education (% high)** | 46.2 | 50.8 | 41.9 |
| **Ethnicity (%white)** | 96.2 | 95.9 | 95.9 |
| **Smoking** |  |  |  |
| ***Current*** | 14.2 | 15.5 | 13.3 |
| ***Former*** | 45.8 | 46.7 | 44.9 |
| ***Never*** | 40.0 | 37.8 | 41.9 |
| **Physical activity (MET-h per week leisure)** | 30.4 (16.0-51.2) | 31.4 (15.8-52.5) | 29.8 (16.5-50) |
| **Energy intake (kJ/day)** | 9475 ± 2904 | 10592 ± 3234 | 8474 ± 2238 |
| **Alcohol intake (g/day)** | 10.4 (2.8 – 21.5) | 16.9 (5.2-27.9) | 7.3 (1.4-14.5) |
| **Glucose (mmol/L)** | 5.3 (5.0-5.7) | 5.4 (5.1-5.9) | 5.1 (4.8-5.6) |
| **Isoleucine (µmol/L)** | 50.3 ± 14.7 | 58.1 ± 15.6 | 43.4 ± 10.4 |
| **Leucine (µmol/L)** | 66.0 ± 13.7 | 74.2 ±13.4 | 58.7 ± 9.8 |
| **Valine (µmol/L)** | 153.9 ± 27.6 | 169.4 ± 26.0 | 140.1 ± 21.7 |
| **Phenylalanine (µmol/L)** | 52.6 ± 5.2 | 54.0 ± 5.5 | 51.3 ± 4.6 |
| **Tyrosine (µmol/L)** | 53.0 ± 8.8 | 55.0 ± 9.2 | 51.2 ± 8.1 |
| **Histidine (µmol/L)** | 54.8 ± 6.3 | 55.8 ± 6.9 | 54.0 ± 5.7 |

Participant characteristics in 1983 participants. Results are weighted towards the BMI distribution of the general population, and are expressed as mean ± standard deviation (SD) for normally distributed data or median with interquartile ranges (IQR) for non-normally distributed data. *BMI, body mass index; FM, fat mass; IHL, intrahepatic lipid content; MET, metabolic equivalent of tasks.*

Table 2. Relative change in IHL content and 95% confidence intervals per SD of plasma amino acid level in participants of the NEO study (45 to 65 years), stratified by sex.

|  | **Total**  (n=1 983) | **Male**  (n=1 042) | **Female**  (n=941) | ***P* value for interaction** |
| --- | --- | --- | --- | --- |
| **Isoleucine** | 1.52 (1.43, 1.61)* | 1.34 (1.25, 1.43) | 1.89 (1.72, 2.08) | < 0.001 |
| **Leucine** | 1.54 (1.44, 1.64)* | 1.35 (1.25, 1.46) | 1.85 (1.68, 2.03) | < 0.001 |
| **Valine** | 1.29 (1.21, 1.38)* | 1.18 (1.09, 1.28) | 1.42 (1.29, 1.56) | 0.003 |
| **Phenylalanine** | 1.14 (1.07, 1.21)* | 1.07 (0.99, 1.15) | 1.22 (1.10, 1.34) | 0.032 |
| **Tyrosine** | 1.31 (1.24, 1.39) | 1.27 (1.19, 1.37) | 1.35 (1.24, 1.47) | 0.310 |
| **Histidine** | 1.03 (0.97, 1.09) | 1.01 (0.93, 1.10) | 1.05 (0.97, 1.14) | 0.515 |

Linear regression analysis including fasting plasma BCAA and AAA levels as exposure and log-transformed IHL content as outcome, and were weighted towards the BMI distribution of the general population. Model adjusted for age, sex, total body fat, alcohol and energy intake as well as for leisure time physical activity and include an interaction term between sex and amino acid concentration. The regression coefficients with 95% CI represent relative changes in IHL content per SD of plasma amino acid level. Such ratio, for example 1.2, can be interpreted as 1.2 times IHL content for each extra SD in amino acid concentration, which would reflect an increase in IHL content from, for example, 5% to 6%. Asterisks (*) indicate a significant interaction with sex (p < 0.05).

Table 3. Participant characteristics of the exercise intervention study

|  | **NAFL** (n=7) | **T2DM** (n=7) | **CON** (n=7) |
| --- | --- | --- | --- |
| **Age (years)** | 55.6 ± 5.4 | 63.1 ± 5.8 | 59.0 ± 7.9 |
| **BW (kg)** | 104.7 ± 8.1 | 91.1 ± 5.0 ^*^ | 89.4 ± 5.3 ^*^ |
| **BMI (kg/m^2^)** | 31.3 ± 2.7 | 29.2 ± 2.0 | 29.0 ± 1.7 |
| **FM (kg)** | 30.9 ± 4.5 | 25.6 ± 3.3 ^*^ | 25.4 ± 2.0 ^*^ |
| **FFM (kg)** | 71.0 ± 4.7 | 63.6 ± 3.4 ^*^ | 61.9 ± 3.9 ^*^ |
| **FM (%)** | 29.4 ± 0.03 | 27.8 ± 0.03 | 28.2 ± 0.02 |
| **FFA fasting (μmol/l)** | 786 ± 177 | 666 ± 108 | 671 ± 188 |
| **ASAT (U/l)** | 25.4 ± 4.4 | 25.7 ± 6.7 | 21.7 ± 4.3 |
| **ALAT (U/l)** | 36.3 ± 5.0 | 41.1 ± 16.7 | 28.4 ± 13.0 |
| **GGT (U/l)** | 39.9 ± 12.2 | 38.2 ± 16.4 | 33.0 ± 16.2 |
| **CRP (mg/l)** | 1.51 ± 0.85 | 1.94 ± 1.38 | 1.31 ± 0.77 |
| **VO_2_ max (ml/kg/min)** | 24.7 ± 4.2 | 25.3 ± 3.8 | 28.1 ± 4.6 |
| **Wmax (watt/kg)** | 1.9 ± 0.3 | 1.9 ± 0.3 | 2.2 ± 0.4 |

Participant characteristics (all males) measured before the start of the exercise training program for people with NAFL (n=7), T2DM (n=7) and CON (n=7). Data are expressed as mean ± SD; * *p*< 0.05 vs. NAFL. *BW, body mass; BMI, body mass index; FFM, fat free mass; FM, fat mass; FFA, free fatty acids; ASAT, aspartate-amino-transferase; ALAT, alanine-aminotransferase; GGT, γ-glutamyltransferase; HDL, high density lipoprotein; CRP, C-reactive protein; VO2 max, maximal oxygen uptake; Wmax, maximal Watt output.*

Figure 1


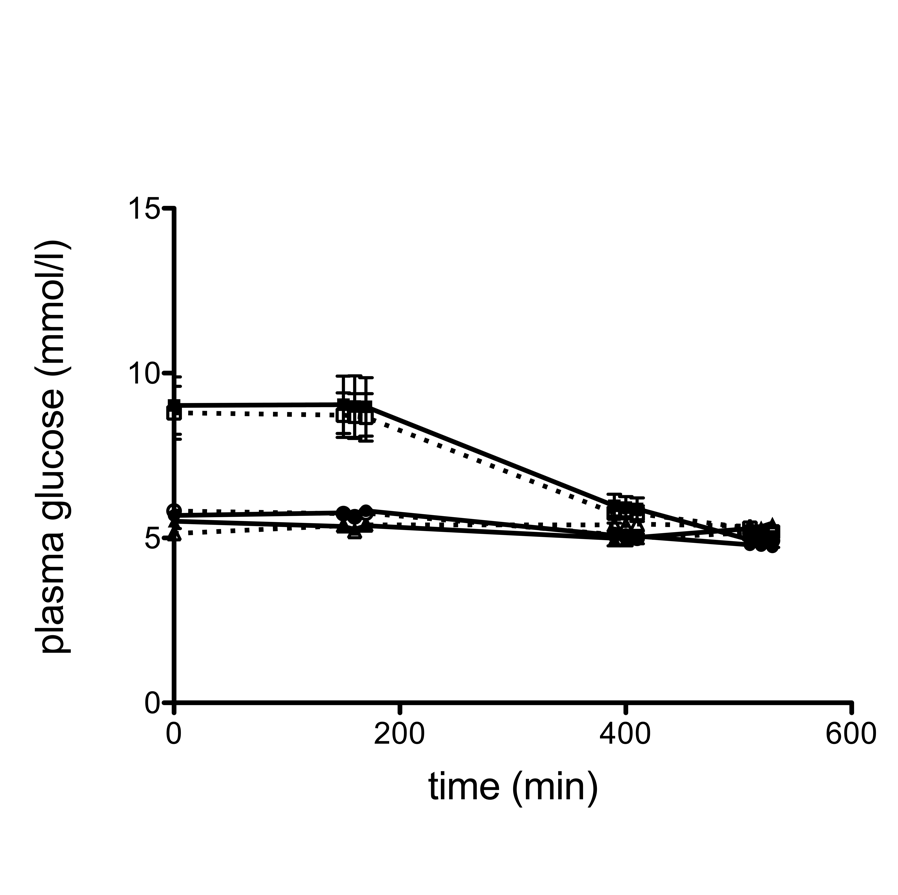


**Fig 1 Plasma glucose values during the steady state phases of the 2-step hyperinsulinemic euglycemic clamp test**. Plasma glucose values at t=0 min, the pre-infusion phase t=150-170 min, low insulin phase t=390-410 min and high insulin phase t=510-530 min. Solid lines represent pre-exercise training and dotted lines indicate post-exercise training program; triangles represent CON, squares represent T2DM and dots represent NAFL. Data are expressed as mean ± SE.

Figure 2


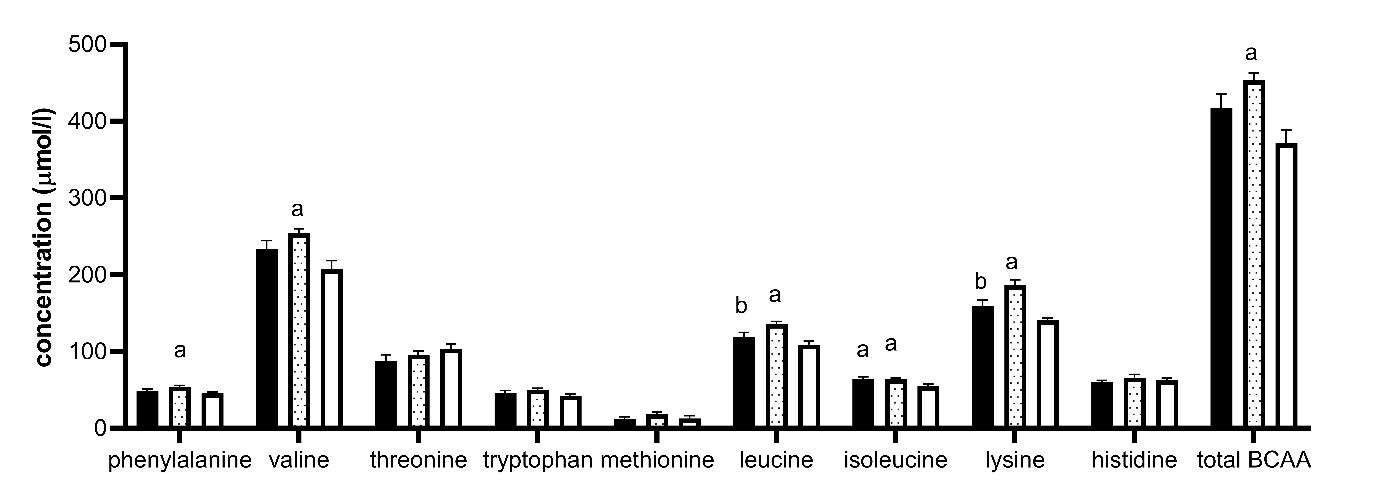
**Fig 2 Baseline fasting essential amino acids levels**. Fasting essential amino acids (μmol/l) measured in plasma before the exercise program in people with T2DM (solid bars, n=7), NAFL (dashed bars, n=7) and in CON (open bars, n=7). Data are expressed as mean ± SE and tested using Anova for repeated measurements; ‘a’ indicates *p*< 0.05 vs. CON and ‘b’ indicates *p*< 0.05 vs. NAFL.

Figure 3


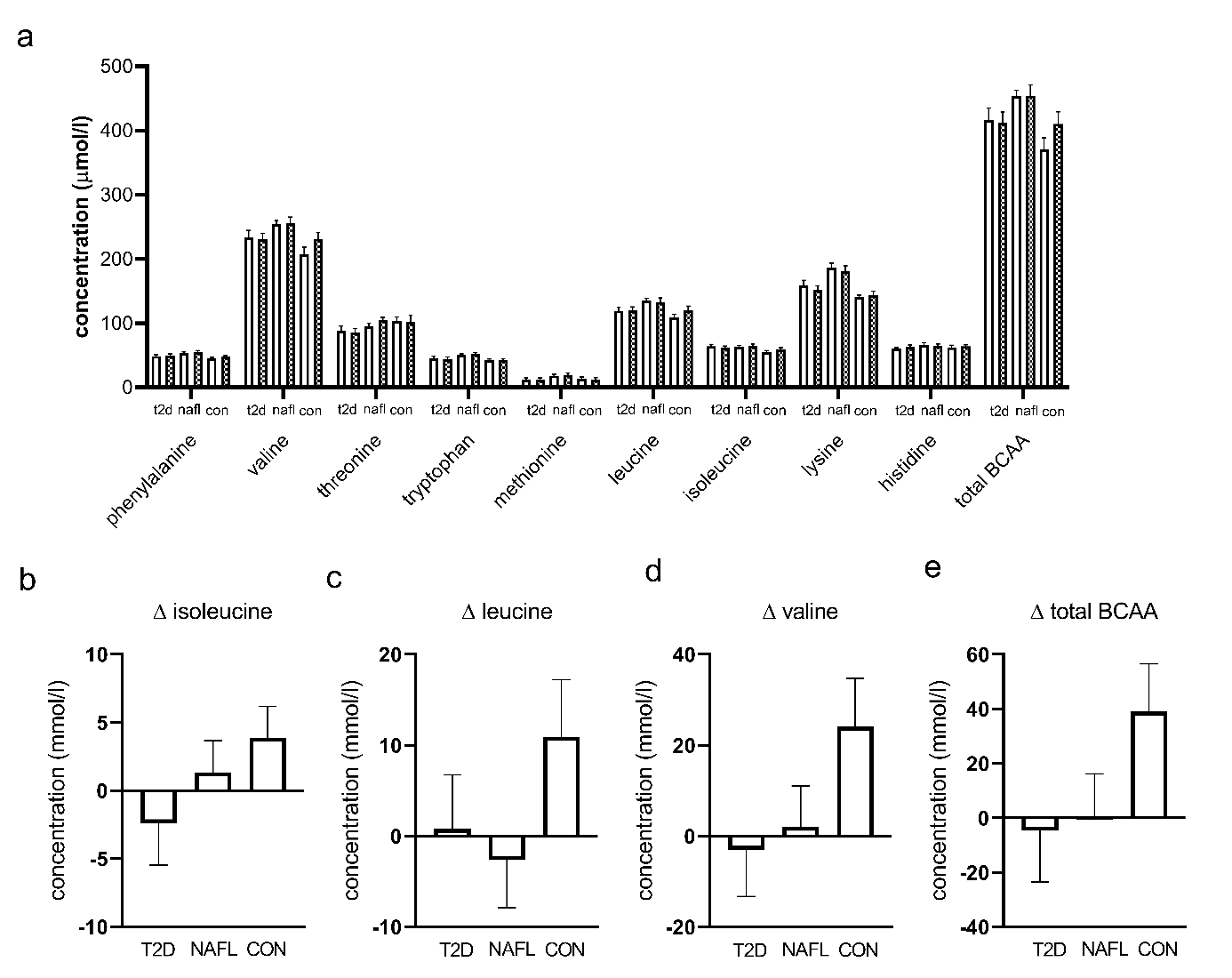


**Fig 3: Changes in fasting essential amino acid levels after exercise training.** Fasting essential amino acids (μmol/l) measured in plasma before (open bars) vs. after (dashed bars) the exercise program (a) in people with T2DM (n=7), NAFL (n=7) and in CON (n=7). The delta changes (absolute values) between post minus pre-exercise were calculated for isoleucine (b), leucine (c), valine (d) and total BCAA (e) and compared between people with T2DM (n=7), NAFL (n=7), CON (bars, n=7). Data are expressed as mean ± SE and tested using Anova for repeated measurements
